# The Chemerin/ChemR23 System Does Not Affect the Pro-Inflammatory Response of Mouse and Human Macrophages *Ex Vivo*

**DOI:** 10.1371/journal.pone.0040043

**Published:** 2012-06-29

**Authors:** Benjamin Bondue, Olivier De Henau, Souphalone Luangsay, Thalie Devosse, Patricia de Nadaï, Jean-Yves Springael, Marc Parmentier, Olivier Vosters

**Affiliations:** 1 Institut de Recherche Interdisciplinaire en Biologie Humaine et Moléculaire (I.R.I.B.H.M.), Faculté de Médecine, Université Libre de Bruxelles, Brussels, Belgium; 2 Euroscreen SA, Brussels, Belgium; 3 Service de Pneumologie, Hôpital Erasme, Université Libre de Bruxelles, Brussels, Belgium; University of London, St George's, United Kindom

## Abstract

Macrophages constitute a major component of innate immunity and play an essential role in defense mechanisms against external aggressions and in inflammatory responses. Chemerin, a chemoattractant protein, is generated in inflammatory conditions, and recruits cells expressing the G protein-coupled receptor ChemR23, including macrophages. Chemerin was initially expected to behave as a pro-inflammatory agent. However, recent data described more complex activities that are either pro- or anti-inflammatory, according to the disease model investigated. In the present study, peritoneal macrophages were generated from WT or ChemR23^−/−^ mice, stimulated with lipopolyssaccharide in combination or not with IFN-γ and the production of pro- (TNF-α, IL-1β and IL-6) and anti-inflammatory (IL-10) cytokines was evaluated using qRT-PCR and ELISA. Human macrophages generated from peripheral blood monocytes were also tested in parallel. Peritoneal macrophages from WT mice, recruited by thioglycolate or polyacrylamide beads, functionally expressed ChemR23, as assessed by flow cytometry, binding and chemotaxis assays. However, chemerin had no effect on the strong upregulation of cytokine release by these cells upon stimulation by LPS or LPS/IFN-γ, whatever the concentration tested. Similar data were obtained with human macrophages. In conclusion, our results rule out the direct anti-inflammatory effect of chemerin on macrophages *ex vivo*, described previously in the literature, despite the expression of a functional ChemR23 receptor in these cells.

## Introduction

As humans are continuously exposed to miscellaneous injuries, inflammation occurs frequently as a result of the activation of protective systems. Higher vertebrates have developed two highly interconnected systems of protection: the innate and adaptive immune systems, for the purpose of eliminating pathogenic insults, removing damaged tissues, and restoring tissue homeostasis [1]. Macrophages constitute a major component of innate immunity and play an essential role in defense mechanisms against external aggressions such as infectious diseases. These properties are mediated in part by their ability to synthesize cytokines and chemokines, which in turn modulate and recruit other leukocyte subsets, linking innate and adaptive immune responses [2]. Moreover, macrophages are also important players in the resolution phase of inflammation, a process considered as passive till recently. Indeed, as a result of their phagocytic properties, macrophages mediate the uptake and clearance of apoptotic cells, necrotic cell debris and wounded tissue components [3].

Chemoattractant agents play a crucial role in the initiation of immune responses, by regulating the traffic and function of leukocyte populations. Their receptors are therefore considered as potential targets for the development of new therapeutic agents, particularly in the fields of cancer and chronic inflammatory diseases. Chemerin is a small protein of 16 kDa isolated and identified from human inflammatory fluids as the natural ligand of the previously orphan G protein-coupled receptor ChemR23 (also termed CMKLR-1), and acting as a chemoattractant agent for leukocytes expressing this receptor [4]. ChemR23 is expressed by myeloid (mDC) and plasmacytoid dendritic cells (pDC), macrophages and NK cells [4]–[9]. Two additional receptors have been reported to bind chemerin with high affinity, GPR1 [10] and CCLR2 [11], [12], but the biological significance of these receptors in the activities of chemerin is still not known. In contrast, chemerin exerts, through ChemR23, potent chemoattractant properties on leukocyte populations at subnanomolar concentrations [7], [13], [14]. Chemerin is synthesized as an inactive precursor, called prochemerin, present at high concentration in plasma. Prochemerin can be rapidly converted into a full ChemR23 agonist by proteolytic removal of a carboxy terminal hexa- or heptapeptide. This maturation step is mediated by neutrophil-derived serine proteases (elastase and cathepsin G) and serine proteases of the coagulation and fibrinolytic cascades, released or activated as a result of tissue injury, inflammation or infection [13], [15], [16]. Chemerin was also described as an adipokine, playing various roles in the regulation of adipocyte differentiation and the metabolism of lipids and carbohydrates [17]–[19].

**Figure 1:**
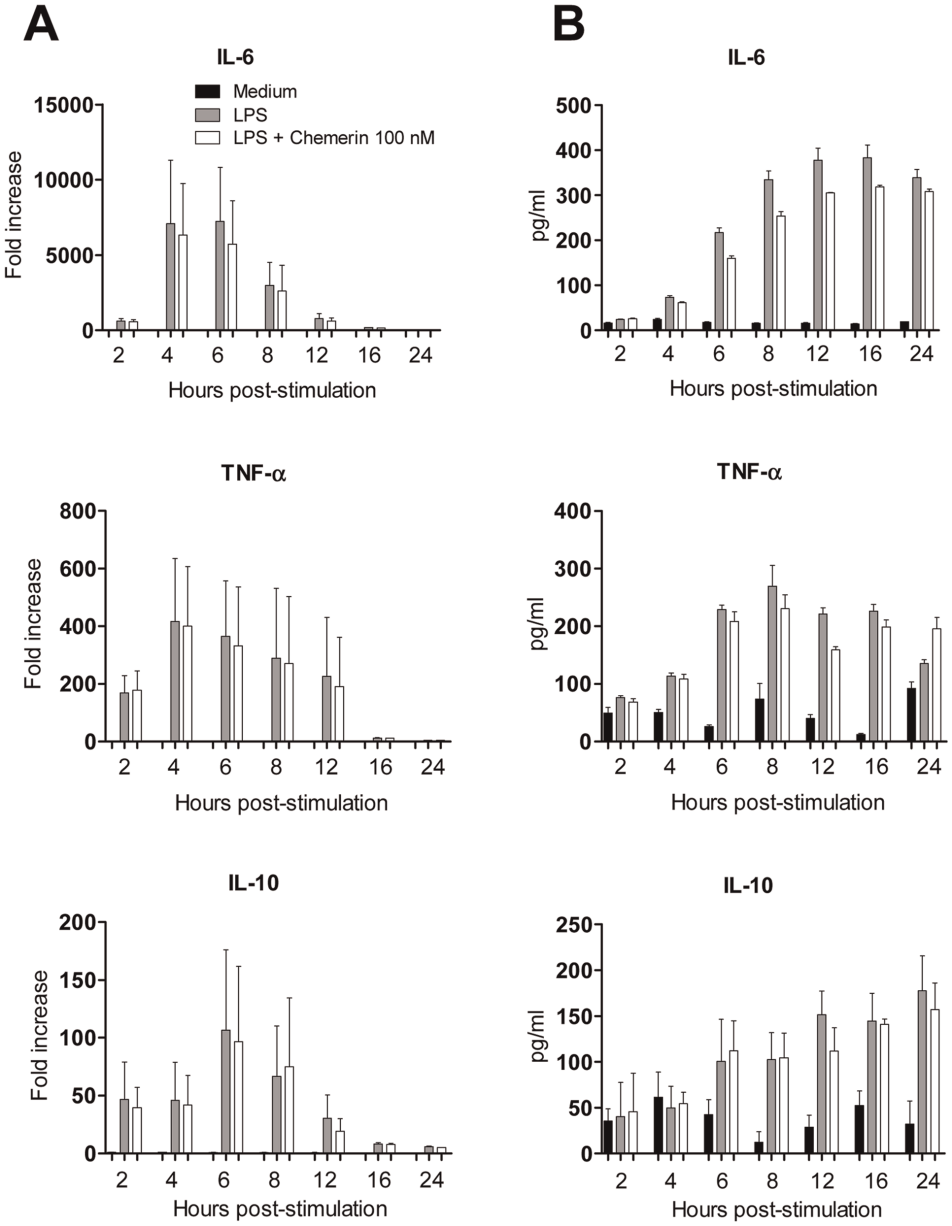
Effect of chemerin on the production of cytokines by activated mouse macrophages. Peritoneal macrophages from WT mice, collected after i.p. thioglycolate injection, were exposed or not for 1 h to 100 nM recombinant mouse chemerin. The cells were further stimulated by 100 ng/ml LPS. Unstimulated cells were used as controls. (A) At the indicated time points, cells were harvested for the determination of transcript levels for IL-6, TNF-α and IL-10, using quantitative RT-PCR. Results are expressed as mean ± SEM for 2 independent experiments performed in duplicate. (B) At the indicated time points, IL-6, TNF-α and IL-10 levels were determined in the supernatants by ELISA. Results are expressed as mean ± SEM for 3 independent experiments performed in duplicate.

In addition to its chemoattractant functions, chemerin was proposed to display anti-inflammatory properties on mouse peritoneal macrophages at low picomolar concentrations, dampening the production of pro-inflammatory cytokines and enhancing the production of anti-inflammatory cytokines by these cells [20]. These observations might have explained our own *in vivo* results showing an anti-inflammatory role of chemerin in a LPS-induced acute lung injury model [7] and in a model of acute viral pneumonia [8]. We therefore studied *ex vivo* the anti-inflammatory properties of chemerin within a large range of concentrations on mouse and human macrophages, but failed to confirm any effects of chemerin on the production of pro- and anti-inflammatory cytokines by these cells following their activation by LPS and/or IFN-γ.

**Figure 2:**
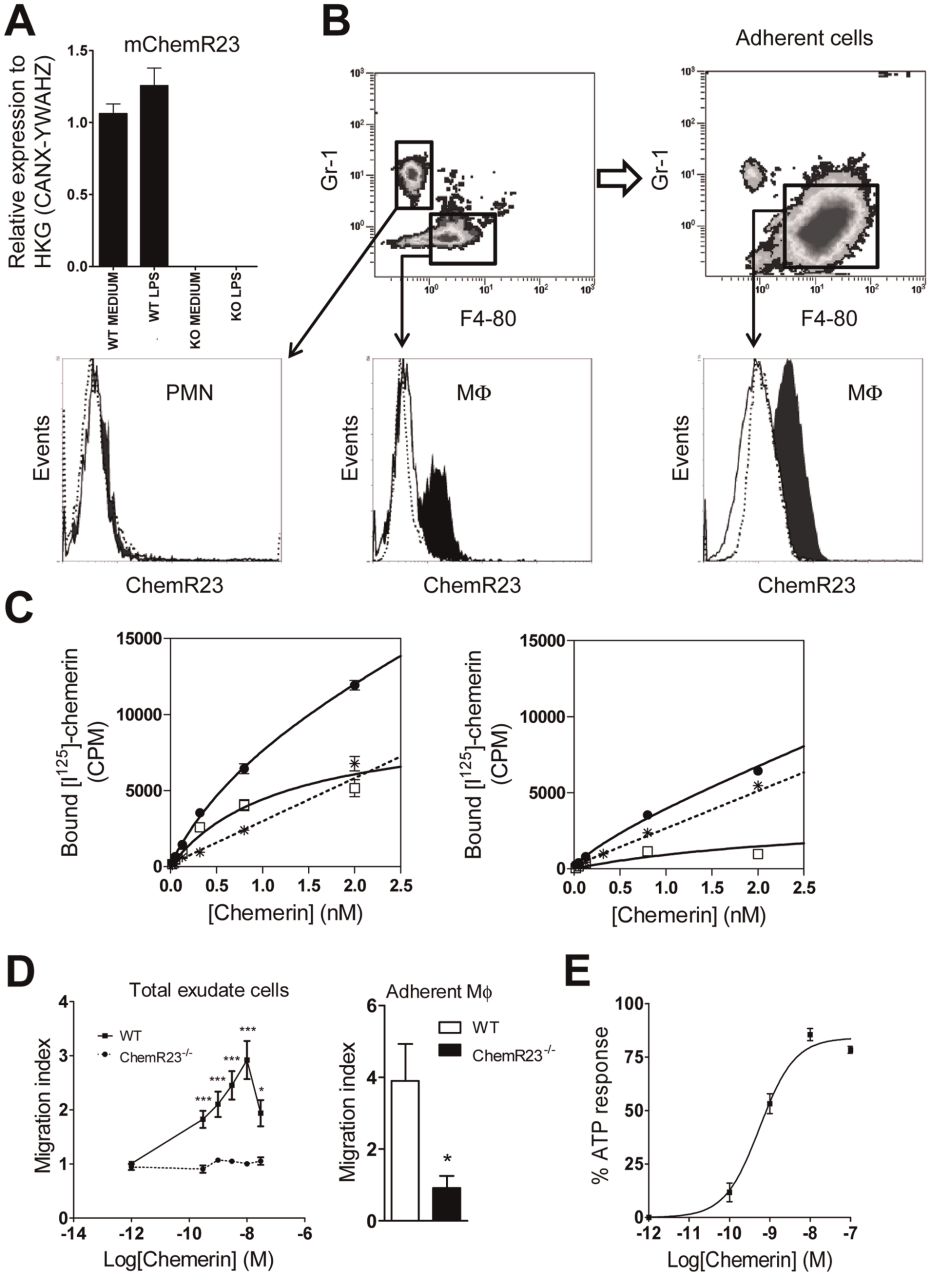
Mouse peritoneal macrophages express a functional ChemR23 receptor. (A) Peritoneal macrophages from WT or ChemR23^−/−^ mice, collected following i.p. Bio-Gel injection, were stimulated for 15 h by 100 ng/ml LPS or left unstimulated. Cells were harvested, and expression of ChemR23 was determined by quantitative RT-PCR. Data are expressed as mean ± SEM for 3 independent experiments performed in duplicate. (B) Peritoneal cells from WT or ChemR23^−/−^ mice, collected following i.p. Bio-Gel injection, were analysed by flow cytometry, directly or after selection by adherence to plates. Cells were stained for leukocyte markers (F4-80 and Gr1) and expression of ChemR23 on F4-80^+^ Gr1^−^ (Mφ) and F4-80^−^ Gr1^+^ (PMN) cells was evaluated using flow cytometry. Histograms represent the fluorescence observed following ChemR23 staining on peritoneal cells from WT (black histogram) or ChemR23^−/−^ (white histogram) mice, compared to the isotype-matched control (dotted line). One representative experiment out of 3 is shown. (C) Total peritoneal cells from WT and ChemR23^−/−^ mice, collected following i.p. Bio-Gel injection, were incubated with increasing concentrations of radiolabeled chemerin (•). Non-specific binding was determined in the presence of a 100-fold excess of unlabeled chemerin (*), and specific binding (□) was calculated as the difference. One representative experiment out of 3 is shown. (D) Peritoneal macrophages from WT or ChemR23^−/−^ mice, collected following i.p. Bio-Gel injection, were tested for their ability to migrate in response to recombinant mouse chemerin. In the left panel (mean ± SEM for 3 independent experiments performed in quadruplicate), the whole populations of cells recovered was used. In the right panel (mean ± SD for an experiment performed in triplicate), macrophages selected by overnight adherence were used. Results are expressed as migration index. (E) The biological activity of mouse recombinant chemerin was measured on mouse ChemR23-expressing CHO-K1 cells using the aequorin-based intracellular Ca^2+^ mobilization assay. Results are expressed as the percentage of the response to ATP and represent the mean ± SD of duplicated data points. One representative experiment out of 3 is shown.

## Methods

### Ethics statement

The experiments using human samples and animals were carried out in strict accordance with the national, European (EU Directives 86/609/EEC) and international guidelines in use at the Université Libre de Bruxelles and in accordance with the Helsinki Declaration. All procedures were reviewed and approved by the local ethic committee (Commission d'Ethique du Bien-Etre Animal, CEBEA) of the Université Libre de Bruxelles (Permit Number: 222N and 341N). All efforts were made to minimize suffering. Written informed consent was obtained from all participants.

**Figure 3:**
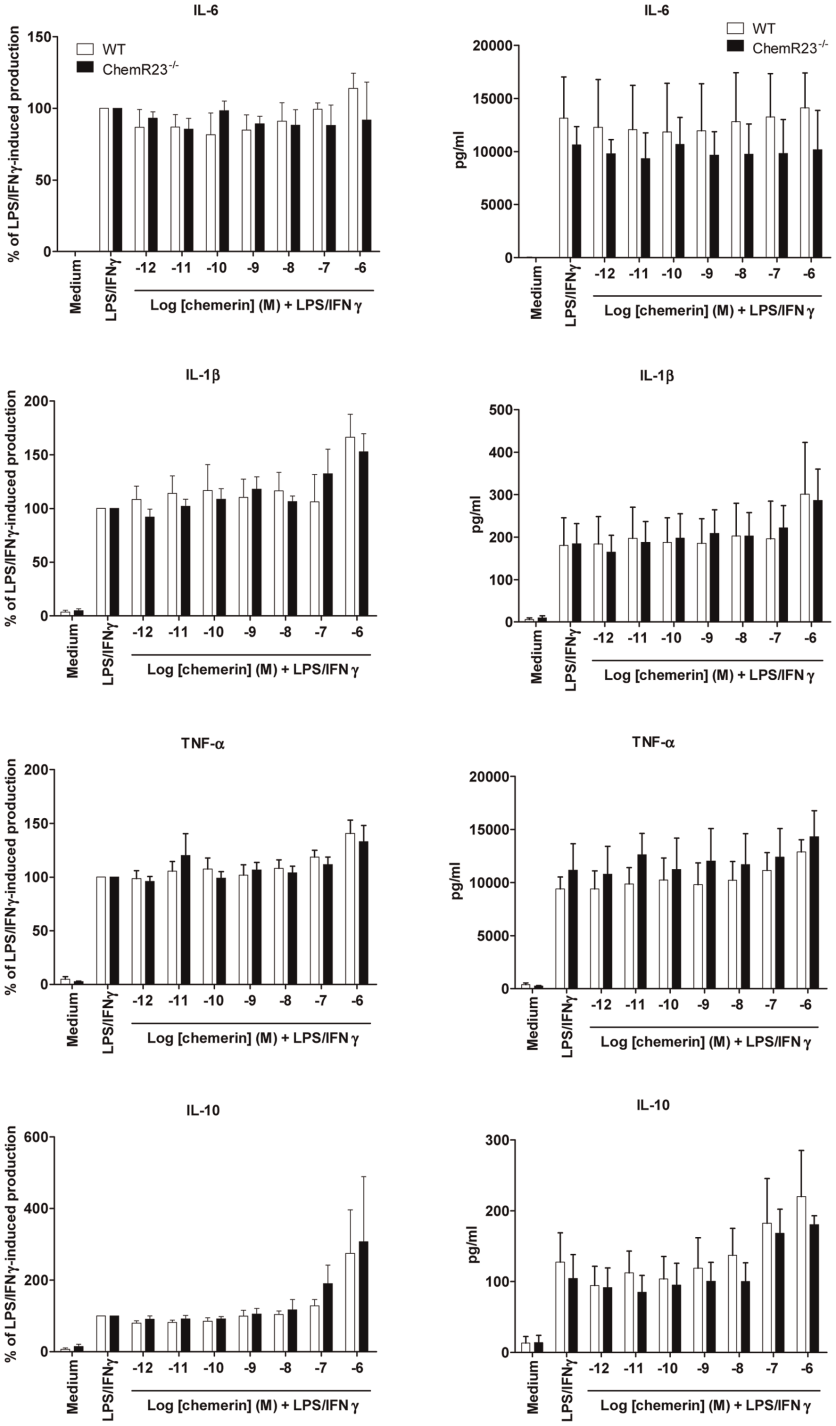
Dose-response effects of chemerin on the production of cytokines by activated peritoneal macrophages. Peritoneal macrophages from WT (white bars) or ChemR23^−/−^ (black bars) mice, collected following i.p. Bio-Gel injection and selected by adherence, were tested for their production of pro-inflammatory (IL-6, IL-1β and TNF-α) and anti-inflammatory (IL-10) cytokines in response to stimulation by LPS and IFN-γ, in the presence or not of graded concentrations of recombinant chemerin (from 10^−12^ to 10^−6^ M). After 15 h of culture, supernatants were collected and cytokine levels were determined by ELISA. Results are expressed as the percentage of the LPS/IFN-γ-induced levels (left panels) or as pg/ml (right panels) and represent the mean ± SEM of 3 to 4 independent experiments.

**Figure 4:**
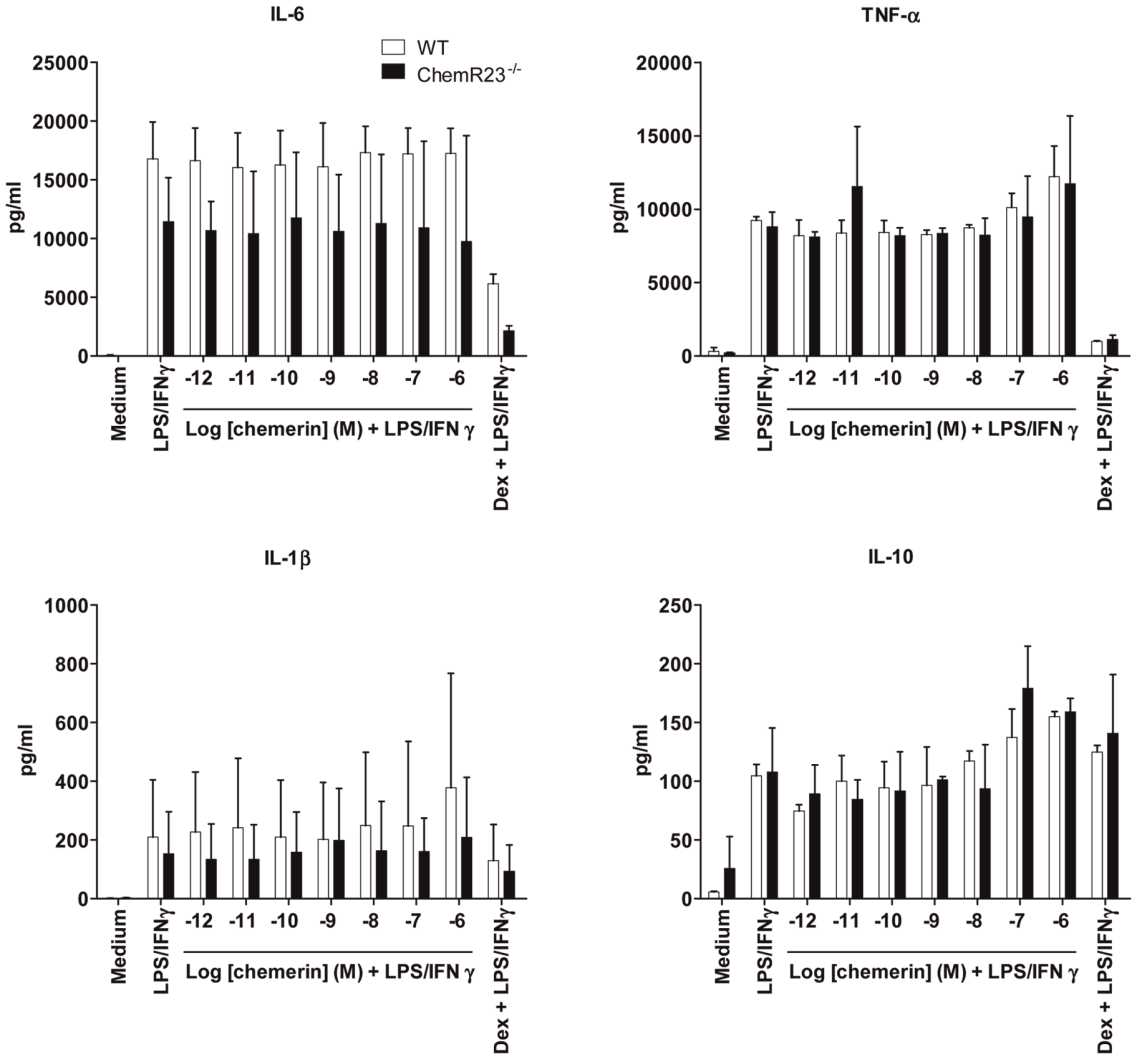
Production of cytokines by activated peritoneal macrophages. Peritoneal macrophages from WT (white bars) or ChemR23^−/−^ (black bars) mice, collected following i.p. Bio-Gel injection and selected by adherence, were tested for their production of pro-inflammatory (IL-6, IL-1β and TNF-α) and anti-inflammatory (IL-10) cytokines in response to stimulation by LPS and IFN-γ, in the presence or not of graded concentrations of recombinant chemerin (from 10^−12^ to 10^−6^ M) or 10^−6^ M dexamethasone (Dex). After 15 h of culture, supernatants were collected and cytokine levels were determined by ELISA. The results, expressed as pg/ml, represent the mean ± SD of 2 independent experiments.

**Figure 5:**
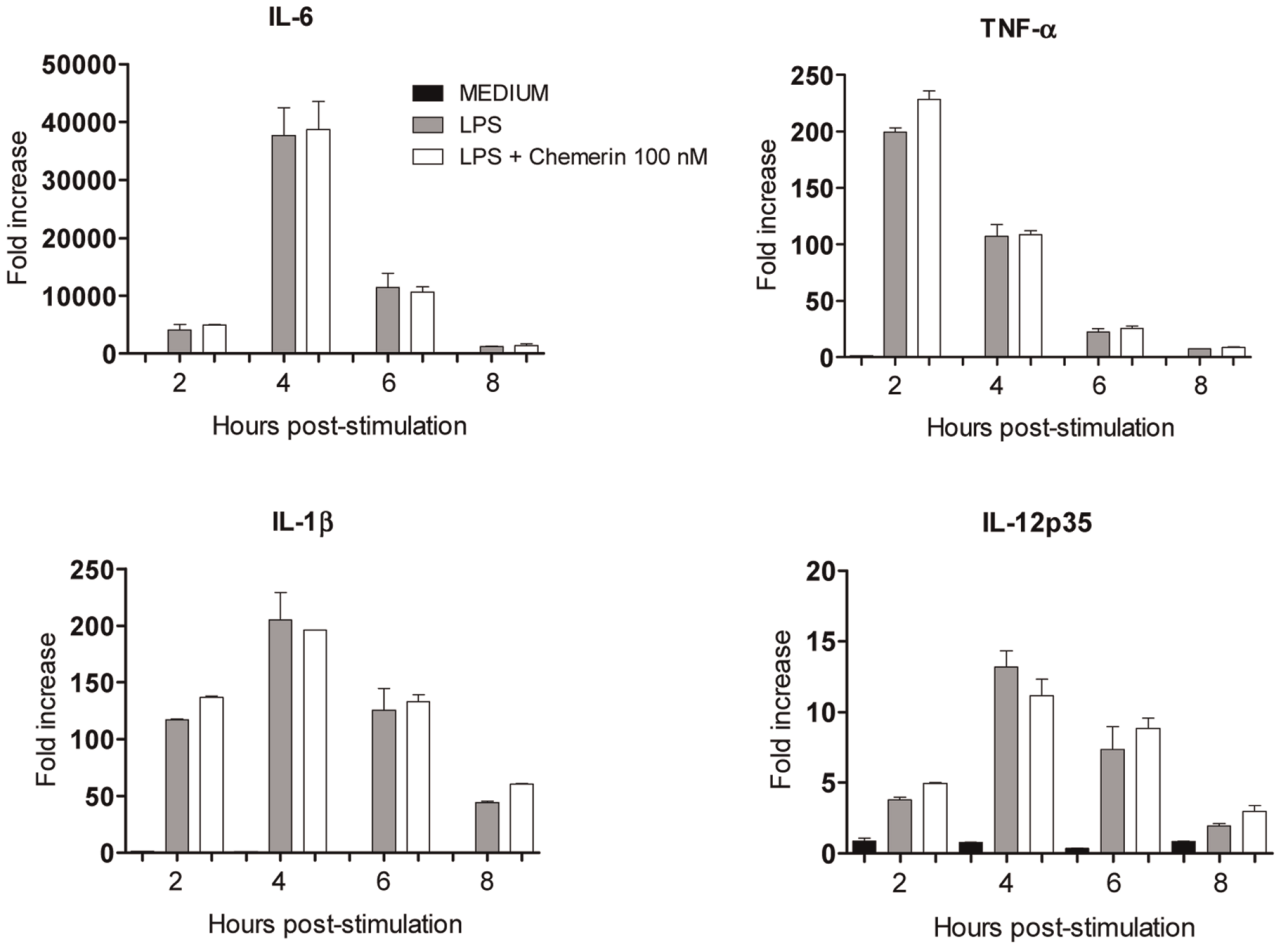
Effects of chemerin on the production of cytokines by stimulated human macrophages. Human macrophages were obtained as described in the Material and Methods section and were tested for their production of IL-6, TNF-α, IL-1β and IL-12p35 following stimulation by LPS, in the absence or presence of 100 nM recombinant human chemerin. At various time points, cells were collected and cytokine transcript levels were determined by quantitative RT-PCR. Results are expressed as fold increase over basal levels and represent the mean ± SEM of 3 independent experiments.

### Animals

Eight to 12-week-old male C57BL/6 mice (Harlan Netherlands) were used throughout the study. ChemR23 knockout (ChemR23^−/−^) mice were obtained from Deltagen and backcrossed for more than 10 generations onto the C57BL/6 background in a specific pathogen-free environment. Heterozygotes (ChemR23^+/−^) were then intercrossed to generate F2 ChemR23^−/−^ mice and their wild-type (WT) controls.

### Mouse peritoneal macrophage preparations

Mouse peritoneal macrophages were collected by using two different protocols. In a first set of experiments, macrophages were harvested 4 days following i.p. injection of a 2% thioglycolate broth. Following sacrifice, the exudate was collected by washing the peritoneal cavity with 2×5 ml of cold PBS (Invitrogen, Gibco, Gent, Belgium). Harvested cells were pooled, centrifuged, washed with PBS (Invitrogen) and resuspended in RPMI 1640 medium (Invitrogen) supplemented with 100 U/ml penicillin (Invitrogen), 100 µg/ml streptomycin (Invitrogen) and 10% heat-inactivated fetal bovine serum (FBS, Invitrogen). Cells were then seeded in 48-well tissue culture plates (Nunc, 4.10^5^ cells in 500 µl) and allowed to adhere for 1 h at 37°C in a humidified atmosphere containing 5% CO_2_. Non-adherent cells were removed, and adherent cells were further used for the macrophage activation assay. The purity of the adherent macrophage population was evaluated by flow cytometry (F4-80^+^ Gr1^−^ cells) to over 90%. In a second set of experiments, WT and ChemR23^−/−^ C57BL/6 mice (5 mice/group) were injected i.p. with 1 ml of a Bio-Gel P100 polyacrylamide bead suspension (2% w/v in PBS, Bio-Rad Laboratories, Nazareth-Eke, Belgium), as described by Cash and colleagues [20]. Four days later, mice were sacrificed and the exudate was collected as above. Washed harvested cells were resuspended in Opti-MEM I medium (Gibco) supplemented with 100 U/ml penicillin and 100 µg/ml streptomycin. The collected cells were either used directly for the chemotaxis assay, binding assay and flow cytometry analysis, or seeded in 48-well culture plates (Nunc, 4.10^5^ cells in 500 µl), and cultured at 37°C in a humidified atmosphere containing 5% CO_2_. Non-adherent cells were removed after 2 or 24 hours by extensive washing and adherent cells were used for the macrophage activation assay and chemotaxis assay, respectively. The population of adherent cells was consistently composed of >90% F4-80^+^ Gr1^−^ macrophages, as determined by flow cytometry analysis.

### Human macrophage preparations

Human macrophages were prepared as previously described [21]. Briefly, leukocytes were recovered from buffy coats of healthy donors on a Ficoll (Lymphoprep Axis-Schield) density gradient and erythrocytes were lysed by ammonium chloride. Monocytes were purified by positive selection using CD14 microbeads (Miltenyi Biotec, Leiden, The Netherlands), were resuspended at the density of 10^6^ cells/ml, and seeded in six-well plates (Nunc) in RPMI 1640 medium (Invitrogen) supplemented with 10% heat-inactivated FBS (Invitrogen). Macrophages were differentiated from monocytes in the presence of recombinant human M-CSF (50 ng/ml, Peprotech, London, UK) for 6 to 11 days. The purity of the cell preparations was evaluated by flow cytometry and over 85% of CD11b^+^/CD206^+^ macrophages were consistently obtained.

### Chemerin binding assay

Chemerin binding assays were performed by incubating 2.10^5^ mouse peritoneal macrophages, harvested after i.p. injection of Bio-Gel polyacrylamide beads, in binding buffer (50 mM Hepes pH 7.4, 1 mM CaCl_2_, 5 mM MgCl_2_, 250 nM sucrose, 0.5% BSA) with different concentrations of radioiodinated recombinant human chemerin (R&D Systems, Abingdon, UK; custom labeling performed by Perkin Elmer, Zaventem, Belgium). Non-specific binding was measured in the presence of a 100-fold excess of unlabeled chemerin. Samples were incubated for one hour and unbound tracer was removed by filtration through GF/B filters pre-soaked in 1% polyethyleneimine. Filters were counted in a γ-scintillation counter. Binding parameters of saturation curves were determined with the PRISM software (Graphpad Softwares) using non-linear regression applied to one site or two sites binding models. The software compared the sum-of-square and the degree of freedom of each regression by using the F test and selected the most appropriate equation.

### Aequorin-based calcium mobilization assay

Functional response of mouse ChemR23 to recombinant mouse chemerin was determined by using an aequorin-based calcium mobilization assay as previously described [13]. Briefly, CHO-K1 cells co-expressing mouse ChemR23, apoequorin and G_α16_ (or control CHO-K1 cells co-expressing apoaequorin and G_α16_), were incubated for 3 h in the dark in DMEM containing 5 µM coelenterazine H (Invitrogen, Molecular Probes). 5.10^4^ cells in a volume of 50 µl were added to wells containing different concentrations of recombinant mouse chemerin and luminescence was recorded for 20 s in a Packard luminometer. Results (as luminescence units) were normalized to the response to 20 µM ATP, and the parameters of the dose-response curves were determined with the PRISM software using nonlinear regression applied to a sigmoidal dose-response model.

### Chemotaxis assay

Chemotaxis assays were performed on peritoneal macrophages (Bio-Gel procedure) as previously described [7]. Briefly, migration of peritoneal macrophages was measured using a 48-well microchemotaxis Boyden chamber with polycarbonate membranes (5 µm pores, NeuroProbe, MD, USA). The cell suspension (5.10^4^ cells in 50 µl) was placed in the upper chamber. The lower wells contained 30 µl of medium with different concentrations of chemoattractant, and the chamber was incubated at 37°C for 90 min. All conditions were tested in triplicate. Controls were performed in the absence of chemoattractant in the lower wells. The results were expressed as migration ratio (mean cell number per well with chemoattractant over mean cell number per well without chemoattractant).

### Macrophage activation assay

Murine macrophages obtained by the thioglycolate procedure were preincubated for 1 h with 10^−6^ to 10^−12^ M recombinant mouse (rm)-chemerin (R&D Systems) in RPMI 1640 medium (Invitrogen), before challenging them with 100 ng/ml LPS (*E. coli* strain 055:B5; Sigma-Aldrich, Bornem, Belgium). Murine macrophages obtained by the Bio-Gel procedure were preincubated for 1 h with 10^−6^ to 10^−12^ M rm-chemerin (R&D Systems) in Opti-MEM I medium (Invitrogen), before challenging them with 100 ng/ml LPS (*E. coli* strain 055:B5; Sigma-Aldrich) and 20 ng/ml rm-IFN-γ (Peprotec). At selected time points after stimulation, supernatants were harvested for cytokine determination and total RNA was extracted from adherent cells for gene expression analysis. Similarly, human macrophages were preincubated for 30 min with 10^−8^ M of recombinant human chemerin (R&D Systems) and subsequently matured for 2, 4, 6, or 8 h by incubation with 100 ng/ml LPS.

### Cytokine assays

Supernatants of cultured macrophages were harvested and levels of interleukin (IL)-1β (detection limit of 31.2 pg/ml), IL-6 (detection limit of 15.6 pg/ml), IL-10 (detection limit of 31.2 pg/ml) and tumor necrosis factor (TNF)-α (detection limit of 15.6 pg/ml) were determined by enzyme-linked immunosorbent assays (BD Biosciences, Erembodegem, Belgium). In some experiments, total RNA was extracted from cultured macrophages and purified using the RNeasy Mini Kit (Qiagen, Venlo, The Netherlands). After DNase I treatment (Ambion), samples were reverse transcribed into cDNA using random hexamers (Roche, Brussels, Belgium) as primers and the Superscript III polymerase (Invitrogen). RT products were analyzed by quantitative real-time RT-PCR. The sequence of primer pairs used for mouse and human genes is provided as supplemental data (Tab. S1 and S2). Raw data were normalized for each sample using YWHAZ and CANX or GAPDH transcripts as references.

### Flow cytometry analysis

Peritoneal macrophages were incubated for 10 min at 4°C with anti-CD16/CD32 monoclonal antibodies (BD Biosciences) to block non-specific binding to Fc receptors. Then, cells were stained with PE-conjugated anti-mouse CMKLR1 (e-Bioscience, clone bz194, Vienna, Austria), FITC-conjugated anti-mouse F4-80 (AbD Serotec, Dusseldorf, Germany), PerCp-conjugated anti-mouse Gr1 (BD Biosciences) monoclonal antibodies or the corresponding isotype-matched controls (BD Biosciences). All samples were analyzed using a FC500 flow cytometer (Beckman-Coulter).

### Statistical analysis

Statistical analysis was performed using nonparametric t tests or tests for multiple comparisons (Kruskal-Wallis test and the Dunn's multiple comparison post-test), using the GraphPad Prism 5 software. For all tests, a p value ≤0.05 was considered as significant.

## Results

### Chemerin does not inhibit the LPS-induced production of pro-inflammatory cytokines by thioglycolate-recruited peritoneal macrophages

Previous experiments have shown that peritoneal macrophages collected from WT mice following intraperitoneal injection of thioglycolate express a functional ChemR23 receptor [7]. Therefore, to further evaluate the potential anti-inflammatory effect of chemerin reported by others [20], peritoneal macrophages were recovered from WT and ChemR23^−/−^ mice 4 days after intraperitoneal injection of thioglycolate and tested in a time-course experiment. Macrophages were stimulated by 100 ng/ml LPS in the presence or absence of 100 nM rm-chemerin, and the expression and release of cytokines were determined at different time points respectively by quantitative RT-PCR (qRT-PCR) and ELISA. As shown in Figure 1A, qRT-PCR analysis performed 2, 4, 6, 8, 12, 16 and 24 hours after LPS stimulation showed an important time-dependent upregulation of TNF-α, IL-6 and IL-10 transcripts, with a peak observed at 4 to 6 hours for the three cytokines. This LPS-induced upregulation was not affected by the presence of 100 nM chemerin in the medium. At the protein level, LPS-stimulated macrophages released high levels of TNF-α, IL-6 and IL-10 with, as expected, peak values obtained at later time-points (i.e. 8–12 hours) than for the corresponding transcripts. Similarly, no significant differences were observed in terms of cytokine levels between cells treated or not with 100 nM of chemerin (Figure 1B).

### Murine peritoneal macrophages obtained from polyacrylamide beads-treated mice express a functional ChemR23 receptor

As we did not observe any anti-inflammatory activity of chemerin on LPS-stimulated peritoneal macrophages collected after thioglycolate injection, similar experiments were conducted according to the protocol published by Cash *et* *al*. [20], using macrophages collected after intraperitoneal injection of polyacrylamide beads and stimulated by LPS and IFN-γ. Macrophages (F4-80^+^ Gr1^−^ cells) represented about 45% of the total cells harvested, and their purity was increased to over 90% following selection by adherence to plates. The purity of the cell population and the expression of ChemR23 were also evaluated after two plating times (2 or 24 hours) without significant differences (data not shown). The total number of cells and the percentage of F4-80^+^ Gr1^−^ macrophages were not different between WT and ChemR23^−/−^ mice. To validate this protocol, we first confirmed by qRT-PCR the presence of ChemR23 transcripts in the macrophage population prepared from WT mice. We showed also that stimulation by LPS did not significantly affect ChemR23 expression in these cells (Figure 2A). The same observation was made following stimulation by IFN-γ (data not shown). Using flow cytometry, surface expression of ChemR23 was then evaluated on cells collected by peritoneal lavage before and after plating. Using a PE-conjugated anti-mouse ChemR23 mAb, we confirmed the expression of the receptor on macrophages but not neutrophils (F4-80^−^ Gr1^+^ cells), using ChemR23^−/−^ cells as controls (Figure 2B). To further confirm the functional expression of ChemR23 by macrophages, we performed binding studies using radioiodinated chemerin and chemotaxis assays. In saturation binding experiments, chemerin bound to cells at a single binding site with a calculated K_d_ of about 1.2 nM (Figure 2C). This value is similar to those obtained for chemerin binding on CHO-K1 cell lines expressing recombinant mouse or human ChemR23 (1.1 nM and 0.9 nM, respectively) (data not shown). In contrast, very weak chemerin binding was detected on macrophages collected from ChemR23^−/−^ mice, confirming that ChemR23 is by far the main chemerin receptor on these cells. Furthermore, macrophages collected after intraperitoneal injection of polyacrylamide beads and selected by adherence to plates, were also tested for their capacity to migrate in response to chemerin. As expected, the cells were shown to migrate in response to increasing concentrations of chemerin with a typical bell-shaped curve culminating for concentrations around 1 nM (Figure 2D); whereas cells collected from ChemR23^−/−^ mice failed to migrate over background levels in response to chemerin. Taken together, these results demonstrated that peritoneal macrophages collected 4 days after intraperitoneal injection of polyacrylamide beads express a functional ChemR23 receptor. Finally, we assessed whether rm-chemerin used throughout our study was able to promote intracellular calcium release through the ChemR23 receptor. A CHO-K1 cell line expressing mouse ChemR23 was used in an aequorin-based calcium release assay. We determined that rm-chemerin wad indeed able to activate its receptor with an EC_50_ of 0.7 nM (Figure 2E). As a control, no calcium response was observed in wild-type CHO-K1 cells exposed to rm-chemerin (data not shown).

### Chemerin does not inhibit cytokine production following stimulation of mouse polyacrylamide-recruited peritoneal macrophages or human blood-derived macrophages

According to the macrophage activation assay described by Cash et al. [20], peritoneal exudates obtained from polyacrylamide beads-treated WT and ChemR23^−/−^ mice were allowed to adhere for 2 hours. After extensive washing, cells were cultured in OptiMEM medium, in the presence of a range of rm-chemerin concentrations (from 10^−12^ to 10^−6^ M) for 1 hour and then stimulated by LPS/IFN-γ. After 15 h of culture, levels of TNF-α, IL-1β, IL-6 and IL-10 in the supernatants were determined by ELISA. As previously observed for thioglycolate-recruited peritoneal macrophages stimulated by LPS alone, none of the induced cytokines was significantly modulated by the presence of chemerin at any concentration (Figure 3). As a control, peritoneal macrophages were also treated with 1 µM dexamethasone before stimulation by LPS/IFN-γ. As shown in Figure 4, dexamethasone repressed the production of pro-inflammatory cytokines, particularly IL-6 and TNF-α. Finally, human macrophages differentiated from peripheral blood monocytes were also generated to assess the effect of chemerin on their LPS-induced cytokine production. As illustrated in Figure 5, 100 nM of recombinant human chemerin had no effect on the expression of the various cytokines tested. Indeed, TNF-α, IL-1β, IL-10 and IL-12p35 transcript levels, evaluated by qRT-PCR 2, 4, 6, and 8 hours after LPS stimulation, were not significantly affected by the presence of chemerin in the medium, whereas a strong increase was monitored in all cases, with peak values obtained 2 (for TNF-α) or 4 hours (for IL-1β, IL-10 and IL-12p35) after stimulation.

## Discussion

In this study, we demonstrate that bioactive chemerin does not exert any modulation of the cytokine inflammatory response of mouse and human macrophages stimulated by LPS or LPS and IFN-γ, despite the large concentration range tested. Recently, Cash and colleagues reported data suggesting that recombinant chemerin at picomolar concentrations could behave *in vitro* as a potent anti-inflammatory mediator on mouse peritoneal macrophages stimulated by LPS and IFN-γ, in a proteolysis-dependent manner [20]. These *in vitro* observations were somehow in line with our own *in vivo* results showing an anti-inflammatory role of the chemerin/ChemR23 axis in a model of acute lung injury induced by LPS [7] and a model of acute viral pneumonia [8]. We therefore addressed the hypothesis of this putative direct anti-inflammatory effect of chemerin on macrophage populations. Surprisingly, while mouse peritoneal macrophages expressed a fully functional ChemR23 and while recombinant chemerin induced chemotaxis of peritoneal macrophages collected from WT but not ChemR23^−/−^ mice, we were not able to reproduce the *in vitro* anti-inflammatory effects of chemerin. Our first experiments were performed with macrophages recruited to the peritoneal cavity using a different procedure than in Cash's report and macrophages were stimulated in a medium containing fetal calf serum. We therefore performed another set of experiments that were conducted using experimental settings identical to those reported by Cash and colleagues. Macrophages were obtained following injection of polyacrylamide beads and stimulated in a serum-free medium allowing the potential proteolysis of chemerin by macrophage-derived proteases. However, even in these experimental settings, no inhibition of the production of the pro-inflammatory mediators TNF-α, IL-6 and IL-1β, and no enhancement of the production of the anti-inflammatory cytokine IL-10 were found when stimulated macrophages were exposed to murine chemerin in a concentration range from 10^−12^ to 10^−6^ M. We specifically used low picomolar concentrations of chemerin, which are in our hands inactive even on cell lines expressing high levels of recombinant human or mouse ChemR23, because Cash and colleagues reported anti-inflammatory activities for such low concentrations of chemerin. Indeed, such a high potency for chemerin differs considerably from all previous experiments showing that chemerin is active on ChemR23 in the 10^−10^ to 10^−8^ M range and peptides derived from its C-terminus in the 10^−9^ to 10^−7^ M range [4], [13], [14]. We confirmed in the present study that low picomolar concentrations of recombinant chemerin did not trigger any signalling in the sensitive aequorin-based calcium mobilizing assay, which was characterized by a somewhat classical EC_50_ of 0.7 nM. In addition, binding of ^125^I-chemerin on peritoneal macrophages yielded a K_d_ value of about 1.2 nM. Finally, no migration of peritoneal macrophages was observed for low picomolar concentrations of chemerin. These results are in agreement with other previous reports showing that mouse DCs and lung interstitial macrophages express ChemR23 and exhibit chemotactic responses to chemerin, but are not affected by chemerin up to concentrations of 10^−6^ M in their production of cytokines or expression of chemokine receptor following their activation [7]. Discrepancies between our *in vitro* observations and those of Cash and collaborators [20] might in part be explained by the genetic background of the mice used in the respective studies. Throughout our experiments, we used peritoneal macrophages from WT and KO mice generated from the same line backcrossed on the C57BL/6 background for over 10 generations (see Methods section). Cash et al. used C57BL/6 mice for most experiments, which should therefore be comparable, but also in some experiments ChemR23 KO mice in the 129SvEv background. In order to extend our observations to human cells, we also evaluated the activity of recombinant human chemerin on human macrophages differentiated from peripheral blood monocytes and following stimulation by LPS. As for mouse cells, no anti-inflammatory activities were identified for chemerin in this *in vitro* setting.

As a conclusion, our results rule out a direct anti-inflammatory effect of chemerin on macrophages. As a consequence, the anti-inflammatory effects of the chemerin/ChemR23 system observed in our models of viral pneumonia and LPS-induced acute lung injury should be explained by other mechanisms. We presently favor an hypothesis following which chemerin exerts its anti-inflammatory properties through its activity on non-leukocytic cells expressing ChemR23, such as epithelial or endothelial cells. This hypothesis is supported by experiments performed on chimeric mice showing that the anti-inflammatory activity of chemerin is not mediated by bone marrow-derived leukocyte populations [8], as well as data showing high expression of ChemR23 in non-leukocytic lung cell populations [22].

## Supporting Information

Table S1 **Sequence of murine primers used for quantitative RT-PCR.**
(DOC)

Table S2 **Sequence of human primers used for quantitative RT-PCR.**
(DOC)

## References

[1] Martin TR, Frevert CW (2005). Innate immunity in the lungs.. Proc Am Thorac Soc.

[2] Taylor PR, Martinez-Pomares L, Stacey M, Lin H-H, Brown GD (2005). Macrophage receptors and immune recognition.. Annu Rev Immunol.

[3] Serhan CN, Chiang N, Van Dyke TE (2008). Resolving inflammation: dual anti-inflammatory and pro-resolution lipid mediators.. Nat Rev Immunol.

[4] Wittamer V, Franssen JD, Vulcano M, Mirjolet JF, Le Poul E (2003). Specific recruitment of antigen presenting cells by chemerin, a novel processed ligand from human inflammatory fluids.. J Exp Med.

[5] Samson M, Edinger AL, Stordeur P, Rucker J, Verhasselt V (1998). ChemR23, a putative chemoattractant receptor, is expressed in monocyte-derived dendritic cells and macrophages and is a coreceptor for SIV and some primary HIV-1 strains.. Eur J Immunol.

[6] Parolini S, Santoro A, Marcenaro F, Luini W, Massardi L (2007). The role of chemerin in the colocalization of NK and dendritic cell subsets into inflamed tissues.. Blood.

[7] Luangsay S, Wittamer V, Bondue B, De Henau O, Rouger L (2009). Mouse ChemR23 Is Expressed in Dendritic Cell Subsets and Macrophages, and Mediates an Anti-Inflammatory Activity of Chemerin in a Lung Disease Model.. J Immunol.

[8] Bondue B, Vosters O, de Nadaï P, Glineur S, De Henau O (2011). ChemR23 Dampens Lung Inflammation and Enhances Anti-viral Immunity in a Mouse Model of Acute Viral Pneumonia.. PLoS Pathog.

[9] Bondue B, Wittamer V, Parmentier M (2011). Chemerin and its receptors in the leukocyte trafficking, inflammation and metabolism.. Cytokine Growth Factor Rev.

[10] Barnea G, Strapps W, Herrada G, Berman Y, Ong J (2008). The genetic design of signaling cascades to record receptor activation.. Proc Natl Acad Sci USA.

[11] Zabel BA, Nakae S, Zúñiga L, Kim JY, Ohyama T (2008). Mast cell-expressed orphan receptor CCRL2 binds chemerin and is required for optimal induction of IgE-mediated passive cutaneous anaphylaxis.. J Exp Med.

[12] Otero K, Vecchi A, Hirsch E, Kearley J, Vermi W (2010). Nonredundant role of CCRL2 in lung dendritic cell trafficking.. Blood.

[13] Wittamer V, Gregoire F, Robberecht P, Vassart G, Communi D (2004). The C-terminal nonapeptide of mature chemerin activates the chemerin receptor with low nanomolar potency.. J Biol Chem.

[14] Vermi W, Riboldi E, Wittamer V, Gentili F, Luini W (2005). Role of ChemR23 in directing the migration of myeloid and plasmacytoid dendritic cells to lymphoid organs and inflamed skin.. J Exp Med.

[15] Wittamer V, Bondue B, Guillabert A, Vassart G, Parmentier M (2005). Neutrophil-mediated maturation of chemerin: a link between innate and adaptive immunity.. J Immunol.

[16] Zabel BA, Allen SJ, Kulig P, Allen JA, Cichy J (2005). Chemerin activation by serine proteases of the coagulation, fibrinolytic, and inflammatory cascades.. J Biol Chem.

[17] Goralski KB, McCarthy TC, Hanniman EA, Zabel BA, Butcher EC (2007). Chemerin, a novel adipokine that regulates adipogenesis and adipocyte metabolism.. J Biol Chem.

[18] Bozaoglu K, Bolton K, McMillan J, Zimmet P, Jowett J (2007). Chemerin is a novel adipokine associated with obesity and metabolic syndrome.. Endocrinology.

[19] Roh SG, Song SH, Choi KC, Katoh K, Wittamer V (2007). Chemerin-a new adipokine that modulates adipogenesis via its own receptor.. Biochem Biophys Res Commun.

[20] Cash JL, Hart R, Russ A, Dixon JP, Colledge WH (2008). Synthetic chemerin-derived peptides suppress inflammation through ChemR23.. J Exp Med.

[21] Devosse T, Guillabert A, D'Haene N, Berton A, de Nadai P (2009). Formyl peptide receptor-like 2 is expressed and functional in plasmacytoid dendritic cells, tissue-specific macrophage subpopulations, and eosinophils.. J Immunol.

[22] Demoor T, Bracke KR, Dupond LL, Plantinga M, Bondue B (2011). The role of ChemR23 in the induction and resolution of cigarette smoke-induced inflammation.. J Immunol.

