# Supplementary material for: The Chemerin/ChemR23 System Does Not Affect the Pro-Inflammatory Response of Mouse and Human Macrophages Ex Vivo
Source: PLoS One. 2012 Jun 29;7(6):e40043. doi: 10.1371/journal.pone.0040043 (PMC3386906; doi:10.1371/journal.pone.0040043)
Supplement: Table S1 — Sequence of murine primers used for quantitative RT-PCR. (DOC) [file pone.0040043.s001.doc]

**Table S1. Sequence of murine primers used for quantitative RT-PCR.**

| Genes Primer sequences  ChemR23 Forward 5'-CCAACTGCCCCAAGAAGGA-3'  Reverse 5'-AATGCAGGCCAGGCATTTC-3'  TNF-α Forward 5'-ACCCTCACACTCAGATCATC -3'  Reverse 5'-GAGTAGACAAGGTACAACCC -3'  IL-10 Forward 5'-GCCACATGCTCCTAGAGCTG -3'  Reverse 5'-CAGCTGGTCCTTTGTTTGAAA -3'  IL-6 Forward 5'-AGGATACCACTCCCACCAGACC -3'  Reverse 5'-AAGTGCATCATCGTTGTTCATACA -3'  CANX Forward 5'-TTGCTGACTCCTTTGACAGAGG-3'  Reverse 5'-CCACTTTCCATCATATTTGGCA-3'  YWHAZ Forward 5'-TGCAACGATGTACTGTCTCTTTTG-3'  Reverse 5'-CGGTAGTAGTCACCCTTCATTTTCA-3' |
| --- |
