# Supplementary material for: The Chemerin/ChemR23 System Does Not Affect the Pro-Inflammatory Response of Mouse and Human Macrophages Ex Vivo
Source: PLoS One. 2012 Jun 29;7(6):e40043. doi: 10.1371/journal.pone.0040043 (PMC3386906; doi:10.1371/journal.pone.0040043)
Supplement: Table S2 — Sequence of human primers used for quantitative RT-PCR. (DOC) [file pone.0040043.s002.doc]

**Table S2. Sequence of human primers used for quantitative RT-PCR.**

Genes Primer sequences

TNF-α Forward 5'-CCCAGGGACCTCTCTCTAATC-3'

Reverse 5'-ATGGGCTACAGGCTTGTCACT-3'

IL-1β Forward 5'-ACAGATGAAGTGCTCCTTCCA-3'

Reverse 5'-GTCGGAGATTCGTAGCTGGAT-3'

IL-6 Forward 5'-AACAGGTAAAGGCCCACTATGC-3'

Reverse 5'-TGACTTGTCCTGAGACCTGATGT-3'

IL-12p35 Forward 5'-TCACAACGGAGGCGAGGTTCTAAGC-3'

Reverse 5'-CCTCTGCTGCTTTTGACACTGAATG-3'

GAPDH Forward 5'-AGCCACATCGCTCAGAACAC-3'

Reverse 5'-GAGGCATTGCTGATGATCTTG-3'
